# Inequitable Distribution of Global Economic Benefits from Pneumococcal Conjugate Vaccination

**DOI:** 10.3390/vaccines12070767

**Published:** 2024-07-12

**Authors:** Fulgence Niyibitegeka, Fiona M. Russell, Mark Jit, Natalie Carvalho

**Affiliations:** 1 Centre for Health Policy, Melbourne School of Population and Global Health, The University of Melbourne, Carlton, VIC 3053, Australia; natalie.carvalho@unimelb.edu.au; 2 Asia-Pacific Health, Murdoch Children’s Research Institute, Melbourne, VIC 3052, Australia; fmruss@unimelb.edu.au; 3 Centre for International Child Health, Department of Paediatrics, The University of Melbourne, Parkville, VIC 3052, Australia; 4 Department of Infectious Disease Epidemiology, London School of Hygiene and Tropical Medicine, London WC1H 9SH, UK; mark.jit@lshtm.ac.uk

**Keywords:** vaccine pricing, inequitable vaccine uptake, fair prices, pricing policy

## Abstract

Many low- and middle-income countries have been slow to introduce the pneumococcal conjugate vaccine (PCV) into their routine childhood immunization schedules despite a high burden of disease. We estimated the global economic surplus of PCV, defined as the sum of the net value to 194 countries (i.e., monetized health benefits minus net costs) and to vaccine manufacturers (i.e., profits). We further explored the distribution of global economic surplus across country income groups and manufacturers and the effect of different pricing strategies based on cross-subsidization, pooled procurement, and various tiered pricing mechanisms. We found that current PCV pricing policies disproportionately benefit high-income countries and manufacturers. Based on the 2021 birth cohort, high-income countries and manufacturers combined received 76.5% of the net economic benefits generated by the vaccine. Over the two decades of PCV availability, low- and middle-income countries have not received the full economic benefits of PCV. Cross-subsidization of the vaccine price for low- and middle-income countries and pooled procurement policies that would relate the vaccine price to the value of economic benefits generated for each country could reduce these inequalities. This analysis offers important considerations that may improve the equitable introduction and use of new and under-utilized vaccines.

## 1. Introduction

Pneumonia remains one of the leading causes of childhood mortality, responsible for over 700,000 childhood deaths in 2021, with more than 50% of these deaths occurring in sub-Saharan Africa and south-east Asia [1,2,3,4]. While pneumococcal conjugate vaccine (PCV) has substantially reduced childhood morbidity and mortality [4], adoption into Expanded Programs on Immunization (EPI) around the world has been uneven and country income-dependent [5]. PCV was first introduced in high-income countries (HICs) in the early 2000s [5,6]. In 2007, the World Health Organization (WHO) issued a recommendation for all countries to include PCV in their EPI [7]. With financial support from Gavi, the Vaccine Alliance, many low-income countries (LICs) have introduced PCV. Similarly, many countries in Latin America have introduced PCV with support for negotiated prices through the Pan American Health Organization (PAHO). However, by the end of 2021, only 148 out of 194 WHO member states had included PCV in national or subnational immunization programs [8,9], and approximately half (49%) of the global birth cohort had not received all recommended PCV doses by age 5, with the majority of these under-vaccinated children living in low- and middle-income countries [9]. In particular, middle-income countries (MICs) that lack support from Gavi or PAHO and self-procure vaccines have lagged furthest in terms of vaccine introduction despite bearing the majority of the global pneumococcal disease burden [5].

PCV has been found to be cost-effective or cost-saving in most countries [10,11,12]. Nevertheless, alongside cost-effectiveness, considerations around vaccine price, affordability, financing, and financial sustainability are key drivers in decision making regarding the introduction of and sustaining new vaccines into the EPI [13]. Unlike childhood vaccines that have been included as part of the EPI for decades, PCV is an expensive vaccine. A recent report from the WHO’s Market Information for Access to Vaccines (MI4A) initiative reported that the high price of PCV was mentioned by respondents in many MICs as a major barrier to introduction [14]. An affordability analysis conducted by the WHO in 32 non-Gavi, non-PAHO MICs found that adding PCV to the vaccine schedule may be financially challenging in 6 of these countries, where introduction would require an estimated 53–87% increase to the existing immunization budget [14].

To achieve the WHO’s Immunization Agenda 2030 (IA2030) [15], there is an increasing need for strategic pricing policies to ensure vaccine affordability in every country, regardless of income. Current formal pooled procurement policies are available through Gavi, which negotiates the lowest prices in the world for the poorest countries and further subsidizes vaccine purchase costs, and PAHO’s Revolving Fund, which allows countries in the PAHO region to access the second lowest prices. However, self-procuring MICs outside the PAHO region and those ineligible or that have transitioned from Gavi support [16] do not have recourse to these mechanisms. Some still access discounted (but higher) prices through the United Nations Children’s Fund (UNICEF), while other countries self-procure vaccines at prices similar to or sometimes higher than HICs [17]. 

The inequitable PCV uptake across countries is well-known as UNICEF and the WHO publish global vaccine coverage data every year [9]. However, the inequality in net societal value produced by PCV across countries and the extent to which the distribution of this value is influenced by current pricing and procurement policies remain less understood. To our knowledge, no study has explored the economic surplus of PCV. The economic surplus of a new technology or vaccine (which can also be referred to as the net societal value or total social welfare) is the sum of the consumer surplus (net economic benefits retained by consumers, in this case, countries, after paying for the vaccine) and the producer surplus (profits made by producers/manufacturers after recovering the cost of production) [18]. The aims of this analysis were to estimate the global economic surplus of PCV and its distribution using an approach that has been utilized previously for the human papillomavirus (HPV) vaccine [19]. Furthermore, we describe the effect of different pricing strategies on the distribution of economic surplus across country income groups and vaccine manufacturers.

## 2. Methods

### 2.1. Study Design

**Figure 1:**
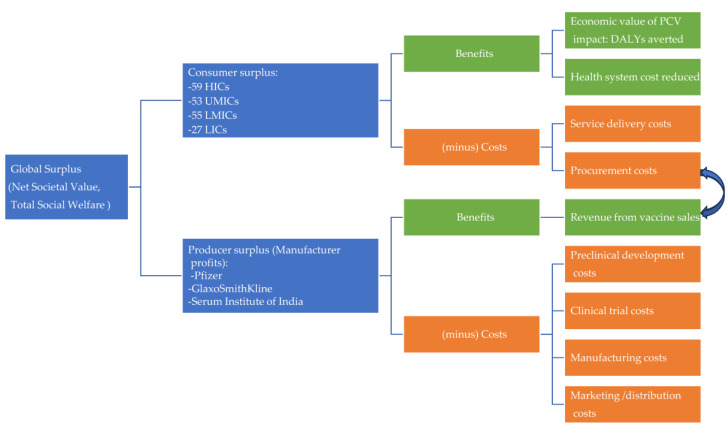
Conceptual framework showing how the global economic surplus for PCV is calculated. **1**. Note: Green boxes indicate benefits and orange boxes indicate costs. 2. Abbreviations: LICs, low-income countries; LMICs, lower-middle-income countries; UMICs, upper-middle-income countries; HICs, high-income countries; DALYs, disability-adjusted life years.

We used a previously defined framework to estimate the net societal value, total social welfare, or global economic surplus of PCV [19]. As illustrated in Figure 1, the global economic surplus consisted of the sum of the net economic benefits to countries as consumers of PCV and the net economic benefits to manufacturers as producers and sellers. The net economic benefit to countries (consumer surplus) was made up of the monetized health benefits of vaccination minus the costs of the vaccination program, while the net benefits to vaccine manufacturers (producer surplus) were calculated as the revenue from PCV sales minus the cost of developing, manufacturing, marketing, and distributing the vaccine. 

To estimate the consumer surplus, we valued benefits and costs for 194 countries and territories grouped by income category—high-income countries (HICs), upper-middle-income countries (UMICs), lower-middle-income countries (LMICs), and low-income countries (LICs)—based on World Bank classification for the year 2021 [20]. For the producer component of economic surplus, we valued benefits and costs for all 3 manufacturers of PCV already prequalified by the WHO [21], i.e., 13-valent PCV manufactured by Pfizer (PCV-13, Prev(e)nar 13^®^), a 10-valent PCV manufactured by GlaxoSmithKline (PCV-10 GSK, Synflorix^®^), and a 10-valent PCV manufactured by Serum Institute of India (PCV-10 SII, Pneumosil^®^), acknowledging that there are other PCVs under development and nationally being licensed for use [21,22].

#### 2.1.1. Consumer Surplus

**Benefits:** Benefits of PCV to the consumer included the monetized value of health benefits measured in terms of disability-adjusted life years (DALYs) averted and healthcare system cost savings resulting from reduced disease burden. To estimate the disease burden reduction by PCV, we used a previously validated model that estimated vaccine effectiveness in terms of incidence rate ratio (IRR) for four clinical outcomes of pneumococcal disease: meningitis (with and without sequelae), pneumonia (invasive and non-invasive), invasive non-pneumonia non-meningitis (NPNM) pneumococcal disease, and acute otitis media (AOM) [10]. We further estimated the total health system cost consisting of the diagnosis, treatment, and services cost to manage each clinical presentation in both the scenario with and without the PCV program. Briefly, the model used a population-based approach and incorporated both the carriage and serotypes coverage data to predict the incidence rate ratios of different clinical presentations of pneumococcal diseases. By using a variety of assumptions, the model condensed the long-term impact projections from more complex susceptible–infectious–susceptible-type dynamic transmission models into a single predictive equation, including serotype replacement and herd immunity. The number of cases of pneumococcal disease and the deaths were estimated by multiplying the expected disease events rate by the IRR adjusted by immunization coverage. The model predictions were based on PCV13-specific serotypes and carriage data. We used interchangeably the PCV impact for both PCV10 and PCV13 and for any dosing schedule assuming non-inferiority across PCVs and dosing schedules, as a recent systematic review found that long-term PCV impact (5 years after PCV10/13 introduction) on pneumococcal disease was similar for PCV10 and PCV13 [23,24]. 

Overall, model input parameters were derived from various sources, including global meta-analysis studies [4,10,25,26], systematic reviews [27,28,29], and electronic databases [30,31] (Table 1). To allow the comparison of the health benefits across countries with different timelines of vaccine introduction, benefits were based on vaccine impact from baseline prior to PCV introduction using country-specific epidemiological disease burden [26]. For simplicity, this analysis was restricted to children under five years; we did not consider the indirect impacts of childhood PCV programs on older age groups or adult vaccination programs. More details about the model, input parameters, and methodological assumptions are provided in the Supplementary Materials. Total DALYs averted were converted into monetary values using country-specific opportunity cost-based thresholds [32]. This approach assumes that PCV programs are government-funded, and funds spent on PCV could have alternatively been directed toward other healthcare programs capable of preventing an equivalent number of DALYs within each country. Other approaches to convert health benefits into economic value were explored in sensitivity analysis, as described below. 

**Cost of PCV program:** The cost of the vaccination program to the consumer was estimated from the provider’s perspective and included the cost of vaccine acquisition and administration. The costs of vaccine acquisition, which included the vaccine purchasing cost and freight cost as well as the cost of injection supplies, were estimated from various sources, including a UNICEF database [33], PAHO database [34], and WHO Market information for access to vaccines (MI4A) database [31]. The delivery cost per dose for any individual country was derived from a recent global modeling study estimating immunization delivery costs across 194 countries [35]. Key model inputs are shown in Table 1, and further details are provided in the Supplementary Materials.

**Table 1:** Model input parameters and sources.

Parameter Description	HICs	UMICs	LMICs	LICs	Source
2021 Birth cohort (million)	12.6	27	70.7	24.2	UN Population Division [30]
Baseline pre-PCV introduction disease burden incidence rate (per 100,0000 children)					
Meningitis	9	13	21	33	O’Brien et al., 2009 [26]
IPD NPNM	52	73	108	166	O’Brien et al., 2009 [26]
Pneumonia	975	1305	2339	3169	O’Brien et al., 2009 [26]
AOM	8984	12,031	14,085	22,330	Monasta et al., 2012 [25]
Mortality rate (per 100,000 children)					
Meningitis	4	8	13	24	O’Brien et al., 2009 [26]
IPD NPNM	3	4	5	7	O’Brien et al., 2009 [26]
Pneumonia	44	77	166	312	O’Brien et al., 2009 [26]
Vaccine impact and effectiveness estimates					
Vaccine impact on IPD < 1 year after vaccination (IRR)	0.63	0.65	0.71	0.74	Chen et al., 2019 [10]
Vaccine impact on IPD > 1 year after vaccination (IRR)	0.53	0.56	0.63	0.67	Chen et al., 2019 [10]
Vaccine impact on non-invasive pneumonia (IRR)	0.80	0.80	0.80	0.80	Canevari et al., 2024 [36]
Vaccine effectiveness against AOM (RR)	0.90	0.90	0.90	0.90	Wannarong et al., 2023 [37]
PCV coverage (2021)	87	39	45	64	WHO/UNICEF [38]
PCV program costs (USD 2021)					
Vaccine delivery cost	18.86	5.94	3.28	1.39	Sriudomporn et al., 2023 [35]
Vaccine price per dose	30.83	16.62	8.99	2.92	UNICEF [33], PAHO Revolving Fund [34], WHO MI4A [31]
Wastage rate (%)	5	5	5	5	UNICEF [33]
Buffer stock (%)	25	25	25	25	UNICEF [33]
Disease management costs (USD 2021)					
Health system cost of pneumonia	3305	1072	481	80	Portnoy et al., 2015; the World Bank; WHO [27,28,29]
Health system cost of meningitis	12,730	6646	2938	668	Portnoy et al., 2015 [27], Chen et al., 2019 [10]
Health system cost IPD NPNM	6111	3497	1417	329	Chen et al., 2019 [10]
Health system cost of AOM	141	51	21	6	Chen et al., 2019 [10]
Health service utilization (%)					
Meningitis	100	100	100	100	Chen et al., 2019 [10]
IPD NPNM	100	100	100	100	Chen et al., 2019 [10]
Pneumonia	100	72	63	53	UNICEF [3]
AOM	85	66	59	54	Chen et al., 2019 [10]
Disability weights					
Pneumonia	0.28	0.28	0.28	0.28	Neonatal pneumonia” in GBD “Mathers et al. 2006 [39]
Meningitis	0.62	0.62	0.62	0.62	Meningitis, S. pneumonia in GBD—Mathers et al. 2006 [39]
IPD NPNM	0.15	0.15	0.15	0.15	Meningococcaemia without meningitis in GBD—Mathers et al. 2006 [39]
AOM	0.013	0.013	0.013	0.013	Otitis media in GBD—Mathers 2006 [39]
Meningitis sequelae	0.06	0.06	0.06	0.06	Meningitis sequelae in GBD—Mathers et al., 2006 (43)
Duration of morbidity (days)					
Pneumonia	7	7	7	7	Ojal et al. [40]
Meningitis	15	15	15	15	Ojal et al. [40]
Meningitis sequelae	Lifetime	Lifetime	Lifetime	Lifetime	Edmond et al. [41], Lucas et al. [42]
IPD NPNM	15	15	15	15	Ojal et al. [40]
AOM	3	3	3	3	Little et al., 2001 [43]

1. Abbreviations: AOM, acute otitis media; IPD, invasive pneumococcal disease; NPNM, non-pneumonia non-meningitis; GBD, global burden of disease; LICs, low-income countries; LMICs, lower-middle-income countries; UMICs, upper-middle-income countries; HICs, high-income countries; IRR, incidence rate ratio; RR, relative ratio; PCV, pneumococcal conjugate vaccine. 2. Note: The parameters in the table are for comparison purposes between the income groups and are aggregated across country-specific estimates used in the model (except vaccine characteristics, wastage, risk of meningitis sequelae, disability weights, and duration of morbidity). Aggregation per income group was performed using the average weighted by the under-five population size except for per capita GDP, where the average was weighted by the total population size in 2021. Effectiveness data for IPD presented by income group is a breakdown of the original predictions, which were presented by regions (see Table S1 of Supplementary Materials).

#### 2.1.2. Manufacturer Surplus

**Manufacturer benefits:** Data on vaccine sales were collected using publicly available annual reports released by each vaccine manufacturer in the United States Securities and Exchange Commission databases or vaccine manufacturers’ companies’ websites [44,45]. We extracted all the revenue made by each vaccine manufacturer from 2000 to 2021. For Pneumosil^®^, we extracted vaccine sales from the WHO MI4A database [17] as it was not reported to SEC. 

**Cost to manufacturers:** The cost of research and development (R&D), including the cost of failure for products that failed to reach market approval, was estimated over the entire development period from preclinical development to post-marketing evaluation based on an approach recently used in previous papers [19,46,47]. The cost of clinical trials was estimated based on the number and size of all clinical trials conducted for each vaccine. A literature review was conducted to identify all PCV-related phase I, II, III, and IV clinical trials funded/sponsored by each manufacturer or subsidiary (see details in the Supplementary Materials). Estimated total R&D cost was later compared with the total revenues made from the first year (2000) of PCV entry to the market up to 2021 to evaluate the manufacturer’s return on investment [45]. A positive return on investment indicated a profitable investment in vaccine development. We further annualized the development cost, assuming a gradual and progressive recovery of the investment over the entire period of patent protection expiring in 2026 [45]. The total cost to manufacturers was therefore estimated as the sum of annual R&D cost and the cost of manufacturing, marketing, and distributing the total number of PCV doses required to vaccinate the 2021 birth cohort in each scenario of the analysis. Further details of the methods to estimate each cost component from the manufacturer’s perspective are provided in the Supplementary Materials. 

### 2.2. Analysis

All cost estimates were converted to 2021 US dollars (USD). Estimated revenues were inflated to 2021 USD using the respective consumer price indexes. In the base case analysis, a discount rate of 3% was applied to future costs and health outcomes as per WHO vaccine evaluation guidelines [48]. We relied on coverage rates achieved by individual countries in 2021 to estimate the current global social welfare and distribution of economic surplus across country income groups and manufacturers. Additionally, we conducted a hypothetical scenario analysis to explore the potential outcomes if at least 90% of the birth cohort across all countries was vaccinated to align with the Immunization Agenda 2030 (IA 2030) global target [15]. Finally, we explored the impact of various alternative pricing scenarios (described below) on the distribution of economic surplus, assuming the IA 2030 coverage target was met.

#### 2.2.1. Sensitivity Analysis

We explored the effect of changes to base case parameters and assumptions. First, we explored the effect of different approaches to monetize health benefits: using a human capital-based approach (1xGDP per capita per DALY averted) and the full income approach, adopted in 2013 by a Lancet Commission in “Global Health 2035” (2.3 times GDP per capita per DALY averted) [49]. The human capital approach is based on the impact of improved health on productivity and earnings and quantifies the economic value of health by considering increased lifespan, reduced medical costs, and higher work productivity. The full income approach extends beyond basic assessments of health relative to per capita gross domestic product (GDP) or foregone earnings and values health by considering both monetary and non-monetary aspects of well-being, acknowledging that health contributes to overall quality of life. More details on the economic valuation of health benefits were previously explored by Herlihy [19]. Second, we looked at how discounting affects our findings. The recent WHO update guide on discounting rates recommends presenting results with two scenarios: one applying a 3% discount rate for both health benefits and costs (which is applied in our base case analysis) and an alternative applying a zero-discount rate to health benefits and a 3% discount rate to costs [50]. We then considered a scenario using a zero-discount rate for health benefits, valuing them the same as present benefits while applying a 3% discount rate to costs. Further, we considered a scenario where future costs and benefits were discounted at a higher rate (5%) each year to reflect values considered in some countries (e.g., Australia) [51]. Third, we explored the effect of uncertainty in vaccine impact estimates as reported in the global modeling study to account for the sparsity of carriage data and heterogeneity of serotype distribution across regions [10]. Hence, we varied vaccine impact on pneumococcal diseases by using the 95% uncertainty interval (lower and upper bounds) from Chen’s study to account for uncertainty related to modeled IRR estimates for vaccine effectiveness [10].

#### 2.2.2. Alternative Pricing Scenario Analysis

We considered the effect of different pricing strategies, including pooled procurement mechanisms, subsidization, and value-based tiered pricing. First, we considered a scenario where all UMICs, LMICs, and LICs access PCV at the Pneumosil^®^ price (USD 1.5) offered to Gavi-eligible countries. Second, we explored a scenario where all Gavi-ineligible MICs would receive the vaccine at the PAHO price (USD 14.14). Third, we explored a scenario based on cross-subsidization of vaccine prices for LICs and LMICs either by HICs or manufacturers. To explore the greatest extent to which this policy could affect the distribution of economic surplus, we explore the most generous but unlikely scenario of zero procurement cost for these countries, either at the cost of HICs or manufacturers. Fourth, we applied the US private market price to self-procurement HICs and UMICs. Finally, we kept the manufacturer’s revenue unchanged and assumed all countries accessed the vaccine at the same price in the absence of any tiered pricing arrangements.

## 3. Results

### 3.1. Manufacturer Return on Investment

From 2000 to 2021, the total manufacturer revenue from PCV sales was USD 91.74 billion (Table 2). Pfizer received the greatest revenue (USD 66 billion) from the sales of Prevnar 13^®^, while its subsidiary Wyeth received the second largest share (USD 17.77 billion) from the sales of Prevnar 7^®^ followed by GSK with revenue of USD 7.88 billion from the sales of Synflorix^®^. Serum Institute of India (SII) received the least revenue of USD 0.09 billion from the sales of Pneumosil^®^. 

As for the cost of PCVs to manufacturers, the results indicate that the total clinical development cost of all pre-qualified PCVs, accounting for the probability of failure in vaccine development, was estimated to be USD 1.4 billion. The cost of the preclinical phase of vaccine development was estimated to be approximately USD 0.6 billion (see Supplementary Table S2). Assuming the total preclinical cost was funded by the vaccine manufacturers, we estimated the total R&D cost to be about USD 2.035 billion. Specifically, the cost of development of PCV was USD 0.50 billion for Prevnar 7^®^, USD 0.77 billion for Prevnar 13^®^, USD 0.73 billion for Synflorix^®^, and USD 0.04 billion for Pneumosil^®^. For the purpose of comparison, from 2000 to 2015, the amount of public funding that was invested in pneumococcal vaccine-related research from preclinical development to public health research was estimated to be USD 857.5 million [52]. This amount represents 339 individual grants awarded to diverse institutions across the globe [52].

Compared with the total R&D cost, manufacturers altogether would have made a return on investment of about 45 times. Similarly to previous studies [19], these findings indicate that the high research and development expenditures can be quickly fully recovered by high revenues from the vaccine sales, particularly to HICs like the United States, where the price of PCV was listed as USD 150.83 per dose in 2021 [53].

### 3.2. Economic Surplus per Vaccinated Cohort

A total of 133.47 million live births were included in the analysis. LICs and LMICs combined represented 71% of the global birth cohort. A total of approximately 5.9 million DALYs were averted in 2021 due to PCVs. The largest share of DALYs averted was in LICs (36.6%) and LMICs (50.0%). PCV was cost-effective in all country income groups when comparing assumed vaccine prices to the average threshold cost in each country income group. The cost per DALY avoided was lowest in LICs and LMICs. Based on the 2021 vaccine uptake, the global economic surplus generated from PCV was estimated to be USD 15.9 billion. HICs and manufacturers received the largest share of the economic surplus at 47.9% and 28.7%, respectively. Of the total manufacturers’ economic surplus, Pfizer received more than 96%. The economic surplus per vaccinated child was estimated to be USD 752 in HICs as compared to USD 120 in UMICs, USD 62 in LMICs, and USD 31 in LICs (Table 3). 

#### 3.2.1. Distribution of Economic Surplus and Sensitivity Analysis

The distribution of the economic surplus among countries and manufacturers varied across different sensitivity analyses. Overall, the share of economic surplus going to LICs remained relatively small, under 6% of the total global surplus (Table 4). This share did not change much when a different monetization rate of DALYs averted was used (either 1 or 2.3 times GDP per capita), with a slightly larger portion of the surplus going to consumers, particularly HICs. Unlike the situation without any discounting, the increased discounting rate reduced the global net consumer benefit to the benefit of the manufacturer. However, the inequitable distribution among consumers (countries’ income groups) remained substantial, disproportionately benefiting HICs. The greatest change in the distribution of economic surplus was observed in the sensitivity analysis on vaccine impact on different serotypes. When the estimated vaccine impact was reduced to the lower bound of its 95% confidence interval, a greater portion of the economic surplus went to manufacturers to the detriment of consumers, particularly those in LMICs; the share of the economic surplus received by LICs was less than 1% in this sensitivity analysis.

#### 3.2.2. Distribution of Economic Surplus under IA2030 Aspirational Coverage Scenario

With the IA2030 coverage target, about 9.1 million DALYs would have been averted in 2021, compared to 5.9 million DALYs using actual 2021 coverage, an additional 3.2 million DALYs averted. Overall, scaling up global PCV coverage to at least 90% would roughly increase the economic surplus to both the manufacturers and consumers, particularly middle-income countries, and would almost double the economic surplus. Globally, the total economic surplus would roughly increase from USD 15.9 billion to USD 20.9 billion. However, HICs and manufacturers would still share more than 68% of the surplus generated by the vaccination (Table 5). This indicates that increasing vaccine coverage without changing vaccine price would have little impact on the inequitable distribution of the economic surplus across country income groups and manufacturers. 

#### 3.2.3. Effect of Pricing Scenarios on the Distribution of Economic Surplus

Overall, the greatest change across alternative pricing scenarios was seen when the full retail price was applied to all self-procurement countries (Table 6). This scenario would dramatically reduce the consumer surplus and increase the manufacturer surplus, with many middle-income countries paying more for vaccination than the value they received in return. If tiered pricing was not used at all, then low-income countries’ share of the surplus would drop. Allocating explicit subsidies to lower-middle- and low-income countries resulted in a notable increase in the surplus for these countries, either at the expense of high-income countries or manufacturers. We found that even with the most generous subsidies, which effectively would eliminate vaccine procurement costs for low- and lower-middle-income countries, the economic surplus captured by high-income countries would only decrease from 47.9% to 27.5%, or by manufacturers from 28.7% to 19.9%. Additionally, if HICs subsidized vaccine costs for LICs and LMICs, then HICs would need to pay up to USD 117.20 per dose, assuming the manufacturer surplus is unchanged.

## 4. Discussion

Our findings indicate that at current PCV pricing and uptake, HICs and manufacturers receive the largest share of the economic surplus generated by PCV. Even at 90% coverage, at current prices, low- and middle-income countries would still receive the lowest share of the economic surplus. These findings are consistent with previous findings for the HPV vaccine [19]. One explanation for this similarity may be that the market supply for both vaccines has been dominated by only two manufacturers (Pfizer and GSK for the PCV and GSK and Merck for the HPV vaccine) based in HICs until recently. Due to a lack of competition, higher prices were set throughout the initial life cycle of the first generation of vaccines before the recent entry into the market of new suppliers based in low- and middle-income countries, such as Pneumosil. Furthermore, even though PCV13 was based on its precursor, PCV7, it came to the market at an even higher price [45]. 

We found even higher levels of inequality in the distribution of economic surplus across country income groups compared to what was estimated for the HPV vaccine. Based on 2015 prices, Herlihy found that per child vaccinated, HICs received two times, four times, and five times the HPV vaccine economic surplus of UMICs, LMICs, and LICs, respectively [19]. Our findings indicate that, based on 2021 PCV prices, HICs received 6 times, 12 times, and 24 times the PCV economic surplus of UMICs, LMICs, and LICs, respectively. 

To reduce this inequity, innovative pricing policies, among other strategies, are required if the full benefits of PCV are to be achieved globally and, in particular, for those with the highest burden. An affordable vaccine price can be achieved through buyer-led (for example, pooled procurement mechanisms or joint efforts to subsidize vaccine purchase cost) or manufacturer-driven (for example, greater tiered pricing) initiatives [54]. In an effort to address the high cost of vaccines and crowding of the current EPI schedule, clinical trials have been completed for PCV10 and PCV13 to evaluate the potential for using two rather than three PCV doses and the use of fractional doses for PCV [55,56,57,58]. These trials have been funded by philanthropy or public funds. Additionally, over the past two decades, there have been several developments in fair vaccine pricing policies and mechanisms, such as Gavi negotiating lower prices for eligible countries via its advance market commitment (AMC) [59], an innovative financing mechanism intended to guarantee a market for pharmaceutical companies for the development of new vaccines. Another example includes the new Gavi MICs strategy adopted in December 2020 to address some key issues related to new vaccine introduction with a focus on PCV, rotavirus vaccine, and HPV in some former Gavi-eligible countries [60]. However, there remains a large, unaddressed gap in providing solutions for equitable pricing and procurement [61] for MICs, some of which have been paying even higher vaccine prices than HICs [17,61]. Despite calls by organizations including Médecins Sans Frontières (MSF) for price reductions [62], median PCV prices increased by 43% from 2019 to 2021 for self-procurement MICs, according to the WHO MI4A report [17]. In recognition of this, the World Society for Pediatric Infectious Diseases has launched a Call to Action for fairer vaccine prices [61]. 

While existing PCV 10 and PCV 13 with WHO prequalification remain underutilized in low- and middle-income countries, HICs are already transitioning to higher-valency PCVs [63,64]. As the use of these higher-valency PCVs increases, the incremental cost-effectiveness of the vaccines will change. Consequently, the model inputs will need to be updated based on the effectiveness of these new vaccines. These extended valency PCVs that are currently in the market in HICs or under development will provide additional protection for up to 25 serotypes. Although the vaccine costs for these are not known, it is highly likely they will be even more expensive, and therefore, the adoption of these vaccines by low- and middle-income countries will be delayed, and this will further drive inequity. 

Our results indicate that the distribution of consumer surplus is more equitable if prices are tiered compared to scenarios without tiered pricing. We found that if all countries paid the same (high) price for PCV, the total consumer surplus would shrink, and many countries, particularly low- and middle-income countries, would be paying more than the value of the vaccine benefits. Our analysis based on 2021 tiered prices indicates that the majority of benefits still favor HICs and manufacturers, highlighting the insufficiency of current tiered pricing to fully address the existing inequitable distribution of social welfare from PCV across countries and manufacturers. Additionally, the mechanism by which prices are set for self-procuring countries is unclear [65]. This study implies that one potential solution to achieve equitable distribution of social welfare from PCV is to explicitly set vaccine prices based on the net societal value of the vaccine to different countries.

Furthermore, results indicate that even the tiered pricing offered to LICs by multinational companies was inferior to competitive prices from developing country manufacturers (DMCs). The economic surplus accrued to low- and middle-income countries substantially increased when applying the Pneumosil^®^ price. This suggests that lowering the intellectual property and technological barriers that LMICs to develop and manufacture vaccines may enable lower prices [66]. The recent WHO prequalification of Pneumosil^®^, a 10-valent pneumococcal conjugate vaccine developed by SII in partnership with PATH and the Bill and Melinda Gates Foundation, has resulted in LMICs being able to access lower PCV prices [67]. In 2023, Pneumosil^®^ was available for LICs at a price of USD 1.5, compared to USD 2.75 for Prevnar 13^®^ and USD 2.9 for Synflorix^®^ after both being available on the market for more than 13 years [33]. 

Nevertheless, recent years have seen progress in reducing vaccine prices, leading many countries to introduce PCV into routine schedules [33]. Additionally, prices of PCV are anticipated to drop further after the entry of new manufacturers based in developing countries into the market and the expiration of patents in 2026 [68]. However, even though generic vaccines and developing country-based vaccines tend to be less expensive [33], their adoption into routine schedules might be delayed due to insufficiency of real-world data to support decision making into EPI, particularly as their counterparts HICs are already moving to high valent vaccines. Delays in adopting affordable PCVs can cause preventable deaths and disabilities. A more steeply tiered pricing policy for already existing vaccines would improve the distribution of the net societal value across countries faster rather than relying solely on new market dynamics.

Findings from this study highlight the importance of cross-subsidies in removing or alleviating the financial barriers to accessing PCV. We found that increasing subsidies to LICs and LMICs could contribute to achieving equitable prices that could accelerate vaccine uptake in these countries while maintaining high consumer surpluses for HICs and positive surpluses for manufacturers. However, implementing this policy would be challenging as it requires an increase in vaccine prices to wealthier countries and/or a reduction in manufacturer revenues with a potential negative impact on the R&D products pipeline. For example, if HICs subsidized vaccine costs for LICs and LMICs, HICs would need to pay up to USD 117.20 per dose, assuming no change in manufacturer profits. While this price is lower than the price USA was paying in 2021 (~USD 150.83 per dose), it is likely to be higher than many other HICs were paying in the same year.

Our findings emphasize the value of pooled procurement mechanisms for MICs; if Gavi-ineligible non-PAHO MICs accessed PCV at PAHO prices, the economic surplus to these countries would more than double. This policy scenario would also result in an increased surplus for manufacturers. There has been some success in regional pooled procurement outside of Gavi pooled procurement through UNICEF and PAHO’s Revolving Fund [54,69]. For example, in May 2012, three Baltic countries (Estonia, Lithuania, and Latvia) initiated a partnership agreement aimed at pooling pharmaceutical and vaccine procurement [69]. The group was able to secure a reduction in price by 17–25% per immunization course compared to what each individual country had previously spent. Data extracted from the MI4A database [31] indicated that countries involved in pooled procurement mechanisms were able to achieve 42% lower prices than self-procurement for 18 widely used vaccines in MICs in 2022, though savings varied across specific vaccines. Hence, international organizations and governments should explore alternative procurement strategies and promote regional cooperation to encourage these pooled procurement mechanisms [61]. 

There are several limitations of the analysis to note. First, the indirect benefits of childhood PCV on the adult population were not captured, which may lead to an underestimation of the consumer surplus of PCV, particularly in HICs with older populations. Another source of underestimation of the surplus is the use of a healthcare system perspective to estimate healthcare cost savings rather than a societal perspective due to a lack of data to inform the latter. Furthermore, the unknown but potentially beneficial impact of PCV on antimicrobial resistance was not considered. Second, the model used in this analysis assumed that the PCV serotype carriage would be eliminated after immunization, but this has not been observed in many low- and middle-income countries [70,71,72,73]. This may lead to overestimation of vaccine impact and thus economic surplus, especially in low- and middle-income countries with a high force of infection [72]. However, we found that even for the lowest vaccine impact, the distribution of economic benefits across country income groups remained the same, and if a lower vaccine impact was selectively considered in low- and middle-income settings, their share of the global economic surplus of PCV would be even lower than currently estimated. Third, this analysis attributed the entire producer surplus to vaccine manufacturers, but some of these profits might be shared with distributors like wholesalers. Fourth, the cost of research and development borne by manufacturers may have been offset by research grants, funds, and loans from public institutions. This might have led to an underestimation of the manufacturers’ economic surplus. Fifth, due to insufficient data on the cost of adverse events following PCV administration, our analysis did not include these events. This may result in an overestimation of the consumer surplus. However, even if our model could account for these adverse effects, it is unlikely that the results regarding current inequality would change. Sixth, data on disease burden and the economic costs of pneumococcal disease are limited in many parts of the world; hence, our results should be interpreted in the context of the sensitivity analyses we conducted around key parameters. Furthermore, private healthcare in low- and middle-income countries is often unmonitored, complicating accurate calculations of vaccine impact and cost-effectiveness. While these limitations may influence the overall magnitude of the estimated global economic surplus, they are unlikely to have a major impact on the distribution of economic surplus across country groupings and producers, nor influence this study’s conclusions with respect to the impact of different pricing policies.

## 5. Conclusions

In conclusion, this study provides clear evidence that current vaccine pricing policies disproportionately benefit HICs and manufacturers, who receive the highest share of the economic surplus generated by PCV. Unaffordable prices due to limited health budgets and competing healthcare needs, and lack of transparency in price setting have negatively impacted vaccine uptake in MICs. This study offers important lessons for other new vaccines and technologies coming to market. It has taken over two decades before PCV has seen widespread introduction in MICs. Early adoption of appropriate pooled procurement mechanisms, promoting vaccine manufacturing in LMICs and more steeply tiered prices, especially in MICs, could promote greater vaccine access outside Gavi and PAHO members. Evidence from this study can be used to inform pricing policies that facilitate equitable dissemination of new and existing vaccines. 

## Figures and Tables

**Table 2:** Manufacturer revenue (million 2021 USD).

Year	Prevnar 7^®^ (Wyeth)	Prevnar13^®^ (Wyeth-Pfizer)	Synflorix^®^ (GSK)	Pneumosil^®^ (SII)
2021	n/a	5272.00	491.02	86.91
2020	n/a	6124.83	539.60	6.12
2019	n/a	6197.22	633.14	n/a
2018	n/a	6260.95	610.43	n/a
2017	n/a	6191.67	724.19	n/a
2016	n/a	6455.67	768.29	n/a
2015	n/a	7139.60	665.47	n/a
2014	n/a	5109.53	749.60	n/a
2013	n/a	4622.46	736.46	n/a
2012	n/a	4858.94	717.77	n/a
2011	n/a	4405.36	675.53	n/a
2010	n/a	3002.27	424.35	n/a
2009	n/a	362.49	143.64	n/a
2009	1942.56	n/a	n/a	n/a
2008	3429.80	n/a	n/a	n/a
2007	3080.69	n/a	n/a	n/a
2006	2477.21	n/a	n/a	n/a
2005	1905.05	n/a	n/a	n/a
2004	1330.74	n/a	n/a	n/a
2003	1194.34	n/a	n/a	n/a
2002	817.82	n/a	n/a	n/a
2001	1008.16	n/a	n/a	n/a
2000	581.76	n/a	n/a	n/a
**Total revenue by brand**	**17,768.15**	**66,003.01**	**7879.47**	**93.02**
**Total revenue for all PCVs**	**91,743.65**			

Abbreviations: GSK, GlaxoSmithKline; SII, Serum Institute of India; n/a, not applicable; PCV, pneumococcal conjugate vaccine.

**Table 3:** Calculation of share of global economic surplus of PCV accrued to different actors, based on 2021 PCV coverage.

Outcome	Global	Consumer				Manufacturer		
		HICs	UMICs	LMICs	LICs	Pfizer	GSK	SII
Birth cohort (2021) (million)	133.47	11.59	26.99	70.65	24.24	n/a	n/a	n/a
Share of birth cohort (%)	100%	9%	20%	53%	18%	n/a	n/a	n/a
DALYs averted (million)	5.90	0.34	0.45	2.95	2.16	n/a	n/a	n/a
Share of DALYs averted	100.0%	5.8%	7.6%	50.0%	36.6%	n/a	n/a	n/a
Healthcare cost savings (million)	518.59	392.95	68.08	48.04	9.52	n/a	n/a	n/a
Cost of PCV program (million)	7202.62	4991.89	968.90	996.49	245.35	880.78	347.73	66.03
Benefits of the vaccine (million)	18,026.90	12,210.21	2168.96	2933.21	714.51	5272.00	491.02	86.91
Cost per DALY averted	1132.76	13,347.13	2015.87	321.68	109.14	n/a	n/a	n/a
Average threshold (USD/DALY averted)	7601	31,816	6829	1317	326	n/a	n/a	n/a
Economic surplus (million)	15,898.25	7611.28	1268.14	1984.77	478.69	4391.22	143.28	20.87
Share of global surplus (%)	100%	47.9%	8.0%	12.5%	3.0%	27.6%	0.9%	0.1%
Economic surplus per immunized child	232	752	120	62	31	97	6	2

1. Note: Costs, economic benefits, and surpluses are in 2021 USD; n/a: not applicable. 2. Abbreviations: LICs, low-income countries; LMICs, lower-middle-income countries; UMICs, upper-middle-income countries; HICs, high-income countries; DALYs, disability-adjusted life years; GSK, GlaxoSmithKline; SII, Serum Institute of India; PCV, pneumococcal conjugate vaccine.

**Table 4:** Distribution of economic surplus and sensitivity analysis.

Analyses	Global	HICs		UMICs		LMICs		LICs		Manufacturer	
	Value	Value	Share	Value	Share	Value	Share	Value	Share	Value	Share
Birth cohort (2021) (million)	133	12	8.7%	27	20.2%	71	52.9%	24	18.2%	n/a	n/a
Base case	15,898	7611	47.9%	1268	8.0%	1985	12.5%	479	3.0%	4555	28.7%
Sensitivity analysis											
Valuation of health at 1XGDP per capita	26,451	12,932	48.9%	2665	10.1%	5010	18.9%	1288	4.9%	4555	17.2%
Valuation of health at 2.3XGDP per capita	63,603	35,722	56.2%	7301	11.5%	12,756	20.1%	3269	5.1%	4555	7.2%
0% discount rate to health benefits and 3% discount rate to costs	43,314	27,305	63.0%	4253	9.8%	5825	13.4%	1375	3.2%	4555	10.5%
5% discount rate to health benefits and costs	9638	3290	34.1%	536	5.6%	1012	10.5%	244	2.5%	4555	47.3%
Vaccine impact increased (95% CI)	21,080	9854	46.7%	2018	9.6%	3736	17.7%	917	4.3%	4555	21.6%
Vaccine impact reduced (95% CI)	10,511	5136	48.9%	513	4.9%	245	2.3%	61	0.6%	4555	43.3%

1. Note: Values of economic surpluses are in million 2021 USD. 2. Abbreviations: LICs, low-income countries; LMICs, lower-middle-income countries; UMICs, upper-middle-income countries; HICs, high-income countries; GDP, gross domestic product; n/a, not applicable.

**Table 5:** Calculation of share of economic surplus of PCV based on IA2030 aspirational coverage.

Outcome	Global	HICs	UMICs	LMICs	LICs	Manufacturer
Birth cohort (2021) (million)	133.47	11.59	26.99	70.65	24.24	133
Share of birth cohort	100%	9%	20%	53%	18%	n/a
DALYs averted (million)	9.12	0.36	0.87	4.94	2.94	n/a
Share of DALYs averted	100.0%	4.0%	9.6%	54.2%	32.3%	n/a
Healthcare cost savings (million)	704.73	418.90	144.38	128.28	13.17	n/a
Cost of PCV program (million)	10,505.22	5346.39	2399.24	2418.27	341.32	2000.76
Benefits of the vaccine (million)	24,511.80	13,059.80	4834.17	5643.33	974.50	8231.69
Cost per DALY averted	1074.64	13,532.34	2581.88	463.62	111.50	n/a
Average threshold (USD/DALY averted)	7601	31,816	6829	1317	326	n/a
Economic surplus (million)	20,942.24	8132.31	2579.31	3353.34	646.35	6230.93
Share of global surplus (%)	100%	38.8%	12.3%	16.0%	3.1%	29.8%
Economic surplus per immunized child	173	763	106	52	30	52

1. Note: Costs, economic benefits, and surpluses are in 2021 USD. 2. Abbreviations: LICs, low-income countries; LMICs, lower-middle-income countries; UMICs, upper-middle-income countries; HICs, high-income countries; DALYs, disability-adjusted life years; n/a, not applicable; PCV, pneumococcal conjugate vaccine.

**Table 6:** Effect of pricing scenarios on the distribution of economic surplus.

Analyses	Global	HICs		UMICs		LMICs		LICs		Manufacturer	
		Value	Share	Value	Share	Value	Share	Value	Share	Value	Share
Birth cohort (2021) (million)	133	12	8.7%	27	20.2%	71	52.9%	24	18.2%	n/a	n/a
**Economic surplus (million USD)**											
Base case	15,898	7611	47.9%	1268	8.0%	1985	12.5%	479	3.0%	4555	28.7%
Hypothetical IA2030 coverage achieved	20,942	8132	38.8%	2579	12.3%	3353	16.0%	646	3.1%	6231	29.8%
**Pricing scenario analysis on top of IA2030 coverage**											
1. All LMICs and LICs pay the tail Gavi price for Pneumosil^®^ (USD 1.5)	21,590	8132	37.7%	4596	21.3%	5044	23.4%	780	3.6%	3038	14.1%
2. All MICs Gavi-ineligible pay the PAHO price (USD 14.14)	21,077	8132	38.6%	3252	15.4%	3476	16.5%	646	3.1%	5570	26.4%
3a. LICs and LMICs fully subsidized by HICs	20,939	5750	27.5%	2579	12.3%	5459	26.1%	920	4.4%	6231	29.8%
3b. LICs and LMICs fully subsidized by manufacturers	21,342	8132	38.1%	2579	12.1%	5459	25.6%	920	4.3%	4251	19.9%
4. Retail price to all self-procurement HICs and UMICs (USD 211.86)	17,445	1621	9.3%	−11,610	−66.5%	3353	19.2%	646	3.7%	23,434	134.3%
5. All countries pay the same price to maintain constant manufacturer surplus (USD 17.02)	20,956	11,689	55.8%	2950	14.1%	748	3.6%	−662	−3.2%	6231	29.8%

1. Note: Values of economic surpluses are in 2021 USD. 2. Abbreviations: LICs, low-income countries; LMICs, lower-middle-income countries; MICs, middle-income countries; UMICs, upper-middle-income countries; HICs, high-income countries; IA2030, Immunization Agenda 2030; n/a, not applicable; PAHO, Pan American Health Organization.

## Data Availability

The data supporting the findings of this study are available within the article/Supplementary Materials. Further inquiries can be directed to the corresponding author.

## Supplementary Materials

The following supporting information can be downloaded at: https://www.mdpi.com/article/10.3390/vaccines12070767/s1, Table S1: PCV impact for invasive pneumococcal diseases per region; Table S2: Estimated research and development cost of PCV. References [3,4,5,7,10,19,21,22,23,24,25,26,27,28,29,30,31,32,33,34,35,37,38,40,41,43,47,65,74,75,76,77,78,79,80,81,82,83,84,85,86,87,88,89,90,91,92,93] are cited in the Supplementary Materials.
