# Supplementary material for: Inequitable Distribution of Global Economic Benefits from Pneumococcal Conjugate Vaccination
Source: Vaccines (Basel). 2024 Jul 12;12(7):767. doi: 10.3390/vaccines12070767 (PMC11281544; doi:10.3390/vaccines12070767)
Supplement: Supplementary file 1 [file vaccines-12-00767-s001.zip › vaccines-3005111-supplementary.pdf]

vaccines

MDPI

Article

Inequitable distribution of global economic benefits from pneumococcal conjugate vaccination

Supplementary material

Description of interventions

The intervention of interest is pneumococcal conjugate vaccine (PCV). The first PCV which included 7 serotypes was manufactured by Wyeth (now owned by Pfizer) and approved by the US FDA in 2000 [1]. Following its successful safety and efficacy profile against pneumococcal diseases, it obtained WHO prequalification in 2007 [2]. It was later replaced by high-valent vaccines and is no longer available on the market. Currently, there are three higher-valent PCVs which have WHO-prequalification: 13-valent PCV manufactured by Pfizer (PCV-13, Prev(e)nar®), 10-valent PCV manufactured by GlaxoSmithKline (PCV-10 GSK, Synflorix®), and 10-valent PCV manufactured by Serum Institute of India (PCV-10 SII, Pneumosil®) [3]. Pneumosil® is the most recent WHO-prequalified vaccine and has not been yet adopted by many countries. There are other 13-valent PCVs approved for use in China and Bangladesh, 14-valent PCV approved for use in India but not yet WHO prequalified [4,5], as well as higher valency PCVs under consideration by high income settings, such as PCV15 and PCV20. Additionally, there are several other high valent PCVs in various stages of development, including PCV 21 (Sanofi/SK, Merck), PCV24 (GSK, Affinivax, 9Bio E, Vaxcyte), PCV25 (InvenThese, Inventrprise), PCV31 (Vaxcyte) and over 10PCV20+ candidates being investigated in China [6]. These vaccines may still need to undergo further trials prior to being qualified. This analysis focussed on childhood PCVs already WHO-prequalified and recommended for the worldwide use.

Estimation of vaccine impact

Vaccine impact estimates were derived from a previous epidemiological global modelling study [7]. Briefly, the model used a population-based approach and incorporated both the carriage and serotypes coverage data to predict the incidence rate ratios of different clinical presentations of pneumococcal diseases. By using a variety of assumptions, the model condensed the long-term impact projections from more complex susceptible-infectious-susceptible-type dynamic transmission models into a single predictive equation, including serotype replacement and herd immunity. This model might not be appropriate for low-income settings where low coverage rates and higher force of infection may prevent these settings from reaching similar indirect effects as HICs. We therefore explored in sensitivity analysis the effect of using the lower and upper bound 95% confidence intervals of vaccine impact estimates as reported in the original study by Chen et al. [7]. The number of cases of pneumococcal disease and deaths were estimated by multiplying the expected disease events rate by the IRR adjusted by immunization coverage.

The model predictions were based on PCV13 specific-serotypes and carriage data. We used interchangeably the PCV impact for both PCV10 and PCV13 and for any dosing schedule assuming non-inferiority across PCVs and dosing schedules, as a recent systematic review found that long-term PCV impact (5 years after PCV10/13 introduction) on pneumococcal disease was similar for PCV10 and PCV13 [8,9].

Results of the vaccine impact for invasive pneumococcal diseases (IPD) used in the model for each region and country are summarised in Table S1. For acute otitis media (AOM), we used pooled estimates of the vaccine efficacy (10.02%) from a recent meta-analysis study that estimate the vaccine efficacy for all-cause AOM [10].

Table S1. PCV impact for invasive pneumococcal diseases per region.

	**<1year after vaccination (IRR)**			**>1 year after vaccination (IRR)**		
	mean	95% CI		mean	95% CI	
**North America**	0.43	0.38	0.47	0.27	0.22	0.33
**Latin America**	0.6	0.55	0.67	0.5	0.43	0.58
**Africa**	0.74	0.58	0.89	0.67	0.47	0.86
**Europe**	0.61	0.54	0.67	0.5	0.42	0.59
**Asia**	0.73	0.58	0.9	0.66	0.47	0.87
**Oceania**	0.64	0.42	0.88	0.55	0.27	0.85

Abbreviation: IRR: incidence rate ratio, CI: confidence interval

Model and data sources

To estimate the total health benefits of the vaccine, we used a previously used and validated decision analytical model that simplified the clinical outcomes in terms of meningitis (with and without sequelae), pneumonia (invasive and non), invasive pneumococcal disease non-pneumonia non-meningitis (NPNM), and acute otitis media (AOM) [7]. The meningitis sequelae was estimated using the risk ratio derived from a previous meta-analysis study [11]. We assumed that 24.8% of pneumonia cases would be invasive based on previous study that estimated the fraction of pneumococcal pneumonia that is bacteraemic [12].

Overall, model inputs parameters were estimated from various sources including global meta-analysis studies [7,13–15], systematic reviews [16–18], and electronic databases [19,20]. Specifically, demographic data such as birth cohort, population under five years, population of children surviving by the age of one year, mortality rate and life expectancy were extracted from the United Nations, Department of Economic and Social Affairs, Population Division [19]. Disease burden data such as incidence of pneumococcal disease and deaths related per each country and region were estimated from the recent global modelling studies on the burden of pneumococcal disease [13–15]. As PCV was introduced at different time in each country, to allow consistent comparison of the economic surplus across countries, we used pre-vaccine introduction morbidity and mortality rates estimates for each clinical endpoint.

DALYs calculation

To calculate the number of years of healthy life lost due to disability (YLDs), we multiplied the total number of cases by condition specific disability weights and duration. Disability weights were obtained from the global burden of disease study [21]. Length of morbidity were obtained from previous studies [22–24]. Age-specific life expectancies were used to estimate the number of years of life lost from mortality (YLLs) due to death from the disease [19].

Cost measurement

Treatment costs

The cost of pneumococcal disease management was estimated from the provider perspective. Therefore, we included only direct medical costs. Various systematic studies and electronic databases [16–18] were used to estimate the cost per episode for each pneumococcal clinical presentation. Chen and collaborators fitted a multiple linear regression model and formulated different equations as function of GDP per capita to predict the cost for each pneumococcal disease clinical presentation [7]. We therefore updated the reported equations to estimate the cost for each pneumococcal disease clinical

presentation. We converted all costs into United States dollar (US$) and inflated to reflect the 2021 values using consumer price index (CPI) [25].

Healthcare resource utilization

Healthcare resources utilization was determined through previous literature. Similar to a previous study, we assumed that all IPD cases are severe and therefore would consult health facilities for healthcare [7]. The proportions of pneumonia who seek medical care were derived from a UNICEF database for all LICs, LMICs and UMICs [26]. Due to the unavailability of data on health seeking behaviour in HICs, based on previous studies we assume that 85.1% and 100% of patients would seek medical care for AOM and non-invasive pneumonia, respectively [7].

Cost of PCV program

The cost of PCV program was calculated as follows:
Cost of program= (vaccine acquisition cost + injection supplies acquisition cost + vaccine delivery cost) *birth cohort*coverage *number of recommended doses.
In the base case analysis, we derived the vaccine price from a UNICEF database for all Gavi-eligible countries [27]. For PAHO members, pricing data were extracted from PAHO database [28]. For self-procurement MICs and HICs, vaccine market price data were derived from the WHO Market information for access to vaccines (MI4A) database [20]. Due to the anonymization of the database, we grouped data based on country income groups and calculated the average price weighted by the volume of purchased doses within each income group. Country-specific delivery cost per dose was derived from a recent global modelling study estimating immunization delivery costs across 194 countries [29]. We used country specific PCV dosing schedule wherever available. WHO recommends a 3-dose regimen of PCV for all children either in 2+1 schedule or 3+0, we therefore included in the base case analysis three doses for countries without PCV currently in the EPI. The list of countries per product type, dosing schedule, and current status/date of introduction into the NIP was extracted from the International Vaccine Access Centre (IVAC), Johns Hopkins Bloomberg School of Public Health [30]. The number of doses required is adjusted by the buffer stock (assumption of 25% similarly to previous studies) and wastage rate of 5% [7]. Coverage data were extracted from the WHO Global Health Observatory data repository [31]. For countries and territories (Austria, Czechia, Monaco) with PCV into EPI (date and schedule of vaccine introduction confirmed by WHO) but coverage data unavailable from the WHO database, we extracted data wherever available either from the country's Ministry of Health or used regional average coverage data.

Consumer surplus

The total number of DALYs averted were converted to monetary units using country-specific opportunity cost-based thresholds in baseline analysis [32]. The total benefits of the vaccine were computed as the sum of total economic value of DALYs averted and treatment cost avoided using vaccine. The consumer surplus was then calculated as the total benefits minus the cost of implementing the vaccination program.
Consumer surplus: [(DALYs*threshold) + treatment cost averted] -Cost of PCV program.

Cost of vaccination to manufacturers

The cost of vaccine to manufacturers consisted of all expenses incurred by the manufacturers from the preclinical phase to the vaccine marketing. Three main components were therefore included to estimate the total cost of vaccine to manufacturer: cost of vaccine research and development, cost of vaccine manufacturing, and cost of marketing.

Cost of research and development

Cost of discovery or preclinical phase

The preclinical cost includes expenditures related to the discovery research and preclinical development. Costs incurred during this phase is mostly neither categorised nor allocated to specific compounds. Since used resources necessary for cost estimation during the preclinical phase were not reported; we assumed that the cost of preclinical phase shares 30.8 % of the total cost of clinical phase based on previous empirical R&D costing study for vaccines [33,34]. However, most of the preclinical development of pneumococcal conjugate vaccine was conducted by researchers from several institutions including academia, industry, and government agencies [35]. The patent for the development of the seven-valent pneumococcal conjugate vaccine was held Dr. Porter W. Anderson of the University of Rochester [36]. We extracted, from RePORTER database, 3 research grants awarded to Dr Porter W. Anderson by National Institutes of Health (NIH) [37].

Year	Project number	Amount (US$ 2021)
1992	5R01AI017938-12	407,076.66
1993	5R01AI017938-13	423,358.63
1994	N01AI045196-001	310,368.53
Total		1,140,803.81

The University of Rochester later granted an exclusive license for the vaccine to a small biotechnology company called Praxis Biologics. Praxis Biologics later merged with a pharmaceutical company Wyeth, in 1995 [38]. As a result of this merger, Wyeth obtained the exclusive license for the pneumococcal conjugate vaccine. Wyeth later developed and marketed the vaccine under the brand name Prevnar®. In 2009, Wyeth was acquired by Pfizer, which continues to manufacture and sell the pneumococcal conjugate vaccine under the Prevnar® brand name [39].

Cost of clinical trials

The cost of clinical trials was estimated based on the number and the size of all clinical trials conducted until the vaccine approval. Being unaware of the exact number and size of clinical trials that the vaccine developer used for submission to request the vaccine licensure, we conducted a literature review to identify all clinicals trials conducted from Phase I until the vaccine was licensed for use. US clinical trials databases (ClinicalTrials.gov), and International Clinical Trials Registry Platform were used to extract all the trials conducted for PCV.

We included in this analysis all clinical trials that were funded by Pfizer or its subsidiary for Prev(e)nar®, GSK for Synflorix®, and SII for Pneumosil®. Clinical trials that did not include directly include the intervention of interest (Prev(e)nar®, Synflorix®, or Pneumosil®) were excluded.

Data extracted: NCT trial number, title, trial location, number of centres, number of participants, location, dates of trials, intervention, phases, intervention, study design, number of participants, phase and start and primary completion dates. We could not identify any study reporting the cost of PCV trials. Therefore, the unit cost that represents the cost of managing the trial, cost related to clinical procedures performed on each subject, the laboratory testing costs, and the data management costs was derived from the previous study that estimated empirically the cost of clinical trial [40]. Therefore, the cost of each individual clinical trial in phase j was obtained by multiplying the total number of participants by the unit cost per participant plus the fixed cost per trial management:

$Cj = (N_{sj} *Csj) +Cfj$; j=phase I, II, III

N_{sj}, C_{sj} C_{fj} represent the total number of participants for each trial, the variable cost per individual, the fixed cost per trial, respectively. More details about equations used were published elsewhere [41].

All costs were converted into USD, deflated to the year of each trial using CPI-U and capitalised to the 2021 USD using an 9% capitalization rate similar to previous studies on pharmaceutical R&D costing studies [33,42,43]. The capitalization was done in the purpose to account for the opportunity cost of investment in pharmaceutical industry

[33,40,41]. Date of marketing approval was used as the timepoint to capitalize the cost of capital.

Incorporating the probability of success for vaccine development

To account for failed candidate compounds during the process of development of the vaccine, we used pooled estimates of probability of success for infectious diseases vaccines from 2000-2015 from Wong's study [44]. The total cost of research and development of PCV is summarised in the following Table.

Table S2. Estimated research and development cost of PCV.

Vaccine	Number of trials	Number of participants	Original cost estimates before capitalization and inflation	Estimated capitalized cost (USD 2021)
PCV 7® (Wyeth)				
Phase 1	6	413	2,292,267	5,121,924
Phase 2	6	812	1,985,043	3,706,137
Phase 3	7	52,166	127,918,492	278,099,800
Phase 4	7	1,845	6,091,424	8,333,071
Total cost			138,287,226	295,260,932
Adjusted* cost			162,274,335	347,013,969
Preclinical cost			72,226,149	154,451,304
Total R&D cost			234,500,484	501,465,273
Prevnar 13® (Pfizer)				
Phase 1	1	249	117,654	209,017
Phase 2	3	1,170	4,789,427	7,332,416
Phase 3	38	24,913	83,614,143	111,693,620
Phase 4	9	89,787	331,471,238	386,249,626
Total cost PCV13			419,992,462	505,484,679
Adjusted* cost PCV13			437,762,522	529,909,219
Preclinical cost			194,842,278	235,855,548
Total R&D cost PCV13			632,604,800	765,764,767
Synflorix® (GSK)				
Phase 1	2	307	1,736,228	2,962,500
Phase 2	8	5,374	22,328,559	30,843,941
Phase 3	41	89,571	295,639,966	378,933,068
Phase 4	1	850	2,935,944	3,648,387
Total cost			322,640,697	416,387,897
Total adjusted* cost			389,744,509	504,217,808
Preclinical cost			173,470,099	224,420,643
Total R&D cost			563,214,609	728,638,451
PCV 10 (SII): Pneumosil®				
Phase 1	1	34	195,341	342,998
Phase 2	2	460	2,251,628	3,547,269
Phase 3	4	3,694	14,073,973	17,871,781

Total cost	16,520,942	21,762,048
Adjusted cost*	20,603,189	27,468,720
Preclinical cost	9,170,206	12,225,962
Total R&D cost	29,773,395	39,694,682
All PCVs		
Clinical phase cost	1,010,384,555	1,408,609,716
Preclinical phase cost	449,708,732	626,953,457
Total R&D cost	1,460,093,287	2,035,563,173

* Adjusted cost took into account the cost for failed product to reach the market approval
Abbreviations: R&D: research and development; SII: Serum Institute of India

Manufacturing cost

The manufacturing cost depends on the quantity of doses produced, formulation and packaging characteristics. For this analysis, approved multi-dose packaging was adopted as it leads to lower cost. Since manufacturing cost of PCV is not reported by manufacturers, similarly to previous study [41], we used as proxy the tail price given to the Gavi eligible countries. This is based on the claims by the pharmaceutical companies that they are selling the vaccine to poorest countries without margin profit rather than recovering the cost incurred during the production and distribution. For the purpose of comparison, the manufacturing cost of HPV vaccine (Gardasil-4 and Cervarix) was estimated to be US$0.19–$0.32 (2014 USD) per dose for a 10-dose vial for a second set production of at least 15.4 million doses [45]. The cost of manufacturing of conjugate vaccines as estimated in the study by Munira et al. for a production of at least 100 million doses per year was estimated to US$1.99/dose [46]. The cost of Dengue Vaccine production ranged from US$0.51–0.65 (2012 USD) per dose in 10-dose vials provided the production is at least 60 million doses of vaccine per year [47].

Marketing and distribution cost

The marketing cost was assumed to be 9% of the total sales based on previous research to account for the cost of selling, general and administrative expenses (marketing and sales as well as administration and management) [48].

Manufacturer surplus

Manufacturer surplus = Benefit of vaccination to manufacturers – Cost of vaccination to manufacturers.
Benefit of vaccination to manufacturers = Revenue from vaccine sale per dose x number of beneficiary per cohort x Vaccine coverage x number of doses given per vaccinated child
Cost of the vaccination to manufacturer: Vaccine discovery cost + Vaccine R&D costs + Vaccine manufacturing costs + Vaccine marketing costs
Total surplus = Consumer surplus + Manufacturer surplus
The manufacturer return on investment was calculated from the vaccine developer perspective and assessed for a time horizon of 2000-2021.

References

1. Levine OS, Schwartz BA, Beneden CAV, Whitney CG, editors. Preventing pneumococcal disease among infants and young children : recommendations of the Advisory Committee on Immunization Practices (ACIP)2000.
2. World Health Organization. Pneumococcal conjugate vaccine for childhood immunization — WHO position paper = Vaccin antipneumococcique conjugué pour la vaccination infantile — note d'information de l'OMS. Weekly Epidemiological Record = Relevé épidémiologique hebdomadaire. 2007;82(12):93-104.

3. The World Health Organization. Considerations for Pneumococcal Conjugate Vaccine (PCV) Product Choice 2021 [cited 2022 Nov 23]. Available from: https://www.who.int/publications/i/item/considerations-for-pneumococcal-conjugate-vaccine-(pcv)-product-choice.
4. Zhao Y, Li G, Xia S, Ye Q, Yuan L, Li H, et al. Immunogenicity and Safety of a Novel 13-Valent Pneumococcal Vaccine in Healthy Chinese Infants and Toddlers. Frontiers in Microbiology. 2022;13.
5. CGTN. First China-made 13-valent pneumonia vaccine to hit the market [Available from: https://news.cgtn.com/news/2020-01-02/First-China-made-13-valent-pneumonia-vaccine-to-hit-the-market-MVR3v4QTle/index.html#:~:text=The%20approved%20China%2Dmade%20pneumococcal,dollars)%20for%20all%20four%20doses.
6. ISPPD-13, editor The Future of Pneumococcal Conjugate Vaccines: The Need and Challenges for Higher Valency Vaccines. ISPPD-13; 2024.
7. Chen C, Cervero Liceras F, Flasche S, Sidharta S, Yoong J, Sundaram N, et al. Effect and cost-effectiveness of pneumococcal conjugate vaccination: a global modelling analysis. Lancet Glob Health. 2019;7(1):e58-e67.
8. Izurieta P, Scherbakov M, Nieto Guevara J, Vetter V, Soumahoro L. Systematic review of the efficacy, effectiveness and impact of high-valency pneumococcal conjugate vaccines on otitis media. Hum Vaccin Immunother. 2022;18(1):2013693.
9. Bennett JC, Knoll MD. 1173. Changes in Invasive Pneumococcal Disease Incidence Following Introduction of PCV10 and PCV13 Among Children < 5 Years: The PSERENADE Project. Open Forum Infectious Diseases. 2021;8(Supplement_1):S677-S8.
10. Wannarong T, Ekpatanaparnich P, Boonyasiri A, Supapueng O, Vathanophas V, Tanphaichitr A, et al. Efficacy of Pneumococcal Vaccine on Otitis Media: A Systematic Review and Meta-Analysis. Otolaryngol Head Neck Surg. 2023.
11. Edmond K, Clark A, Korczak VS, Sanderson C, Griffiths UK, Rudan I. Global and regional risk of disabling sequelae from bacterial meningitis: a systematic review and meta-analysis. Lancet Infect Dis. 2010;10(5):317-28.
12. Said MA, Johnson HL, Nonyane BAS, Deloria-Knoll M, O′Brien KL, for the AAPBST. Estimating the Burden of Pneumococcal Pneumonia among Adults: A Systematic Review and Meta-Analysis of Diagnostic Techniques. PLOS ONE. 2013;8(4):e60273.
13. Monasta L, Ronfani L, Marchetti F, Montico M, Vecchi Brumatti L, Bavcar A, et al. Burden of disease caused by otitis media: systematic review and global estimates. PLoS One. 2012;7(4):e36226.
14. O'Brien KL, Wolfson LJ, Watt JP, Henkle E, Deloria-Knoll M, McCall N, et al. Burden of disease caused by Streptococcus pneumoniae in children younger than 5 years: global estimates. Lancet. 2009;374(9693):893-902.
15. Wahl B, O'Brien KL, Greenbaum A, Majumder A, Liu L, Chu Y, et al. Burden of Streptococcus pneumoniae and Haemophilus influenzae type b disease in children in the era of conjugate vaccines: global, regional, and national estimates for 2000-15. Lancet Glob Health. 2018;6(7):e744-e57.
16. Portnoy A, Jit M, Lauer J, Blommaert A, Ozawa S, Stack M, et al. Estimating costs of care for meningitis infections in low- and middle-income countries. Vaccine. 2015;33:A240-A7.
17. The World Bank. GDP per capita, PPP (current international $) [cited 2021 January 11]. Available from: https://data.worldbank.org/indicator/NY.GDP.PCAP.PP.CD.
18. World Health Organization. WHO-CHOICE estimates of cost for inpatient and outpatient health service delivery costs [cited 2023 22 January]. Available from: https://www.who.int/teams/health-systems-governance-and-financing/economic-analysis/costing-and-technical-efficiency/quantities-and-unit-prices-(cost-inputs)/econometric-estimation-of-who-choice-country-specific-costs-for-inpatient-and-outpatient-health-service-delivery.
19. United Nations. Department of Economic and Social Affairs, Population Division (2021). *World Population Prospects* [cited 2022 January 07]. Available from: https://population.un.org/wpp/.
20. The World Health Organization. Global Vaccine Market Report 2022 [cited 2023 January 26]. Available from: https://www.who.int/publications/m/item/global-vaccine-market-report-2022.
21. James SL, Abate D, Abate KH, Abay SM, Abbafati C, Abbasi N, et al. Global, regional, and national incidence, prevalence, and years lived with disability for 354 diseases and injuries for 195 countries and territories, 1990–2017: a systematic analysis for the Global Burden of Disease Study 2017. The Lancet. 2018;392(10159):1789-858.
22. Källander K, Hildenwall H, Waiswa P, Galiwango E, Peterson S, Pariyo G. Delayed care seeking for fatal pneumonia in children aged under five years in Uganda: a case-series study. Bull World Health Organ. 2008;86(5):332-8.
23. Ojal J, Griffiths U, Hammitt LL, Adetifa I, Akech D, Tabu C, et al. Sustaining pneumococcal vaccination after transitioning from Gavi support: a modelling and cost-effectiveness study in Kenya. The Lancet Global Health. 2019;7(5):e644-e54.
24. Little P, Gould C, Williamson I, Moore M, Warner G, Dunleavey J. Pragmatic randomised controlled trial of two prescribing strategies for childhood acute otitis media. Bmj. 2001;322(7282):336-42.
25. US Bureau of Labor Statistics. Consumer Price Index [cited 2023 March 21]. Available from: https://www.bls.gov/cpi/.
26. Unicef. Pneumonia [cited 2023 March 20]. Available from: https://data.unicef.org/topic/child-health/pneumonia/.
27. Unicef. Vaccines pricing data [cited 2023 February 25]. Available from: https://www.unicef.org/supply/vaccines-pricing-data.
28. The Pan American Health Organization. Revolving Fund Prices [cited 2023 Jan 26]. Available from: https://www.paho.org/en/revolving-fund.
29. Sriudomporn S, Watts E, Yoon Sim S, Hutubessy R, Patenaude B. Achieving immunization agenda 2030 coverage targets for 14 pathogens: Projected product and immunization delivery costs for 194 Countries, 2021–2030. Vaccine: X. 2023;13:100256.

30. International Vaccine Access Center (IVAC) J, Hopkins, Bloomberg, School, of, Public, Health,. Vaccine introduction: PCV current vaccine intro status [cited 2023 Jan 25]. Available from: https://view-hub.org/map/?set=current-vaccine-intro-status&group=vaccine-introduction&category=pcv.
31. World Health Organization. Global Health Observatory data repository. Pneumococcal conjugate (PCV3) immunization coverage estimates by country [cited 2023 March 12]. Available from: https://apps.who.int/gho/data/node.main.PCV3n?lang=en.
32. Woods B, Revill P, Sculpher M, Claxton K. Country-Level Cost-Effectiveness Thresholds: Initial Estimates and the Need for Further Research. Value Health. 2016;19(8):929-35.
33. DiMasi JA, Grabowski HG, Hansen RW. Innovation in the pharmaceutical industry: New estimates of R&D costs. Journal of Health Economics. 2016;47:20-33.
34. Head MG, Fitchett JR, Newell M-L, Scott JAG, Harris JN, Clarke SC, et al. Mapping pneumonia research: A systematic analysis of UK investments and published outputs 1997-2013. eBioMedicine. 2015;2(9):1193-9.
35. Burdman JR. Vaccine design: the subunit and adjuvant approach: Springer; 2012.
36. Anderson PW, Eby RJ. Immunogenic conjugates of streptococcus pneumonial capsular polymer and toxin or in toxiad. Google Patents; 1994.
37. NIH. Research Portfolio Online Reporting Tools (RePORT) [cited 2023 May 12]. Available from: https://report.nih.gov/report-funding.
38. University of Rochester. Childhood Vaccine with Rochester Roots Recognized [cited 2023 May 13]. Available from: https://www.rochester.edu/pr/Review/V70N1/gazette2.html.
39. Kumar BR, Kumar BR. Acquisitions by Pfizer. Wealth Creation in the World's Largest Mergers and Acquisitions: Integrated Case Studies. 2019:85-99.
40. Chit A, Parker J, Halperin SA, Papadimitropoulos M, Krahn M, Grootendorst P. Toward more specific and transparent research and development costs: The case of seasonal influenza vaccines. Vaccine. 2014;32(26):3336-40.
41. Herlihy N, Hutubessy R, Jit M. Current Global Pricing For Human Papillomavirus Vaccines Brings The Greatest Economic Benefits To Rich Countries. Health Aff (Millwood). 2016;35(2):227-34.
42. Wouters OJ, McKee M, Luyten J. Estimated Research and Development Investment Needed to Bring a New Medicine to Market, 2009-2018. Jama. 2020;323(9):844-53.
43. Harrington S. Cost of Capital for Pharmaceutical, Biotechnology, and Medical Device Firms. The Oxford Handbook of the Economics of the Biopharmaceutical Industry. 2009.
44. Wong CH, Siah KW, Lo AW. Estimation of clinical trial success rates and related parameters. Biostatistics. 2019;20(2):273-86.
45. Clendinen C, Zhang Y, Warburton RN, Light DW. Manufacturing costs of HPV vaccines for developing countries. Vaccine. 2016;34(48):5984-9.
46. Munira SL, Hendriks JT, Atmosukarto II, Friede MH, Carter LM, Butler JRG, et al. A cost analysis of producing vaccines in developing countries. Vaccine. 2019;37(9):1245-51.
47. Mahoney RT, Francis DP, Frazatti-Gallina NM, Precioso AR, Raw I, Watler P, et al. Cost of production of live attenuated dengue vaccines: A case study of the Instituto Butantan, Sao Paulo, Brazil. Vaccine. 2012;30(32):4892-6.
48. Kornfield R, Donohue J, Berndt ER, Alexander GC. Promotion of Prescription Drugs to Consumers and Providers, 2001–2010. PLOS ONE. 2013;8(3):e55504.
